# Trichophycin A, a Cytotoxic Linear Polyketide Isolated from a *Trichodesmium thiebautii* Bloom

**DOI:** 10.3390/md15010010

**Published:** 2017-01-06

**Authors:** Matthew J. Bertin, Paul G. Wahome, Paul V. Zimba, Haiyin He, Peter D. R. Moeller

**Affiliations:** 1 Department of Biomedical and Pharmaceutical Sciences, College of Pharmacy, University of Rhode Island, 7 Greenhouse Road, Kingston, RI 02881, USA; 2 Biosortia Pharmaceuticals, Hollings Marine Laboratory, 331 Fort Johnson Road, Charleston, SC 29412, USA; pwahome@biosortia.com (P.G.W.); haiyn_he@yahoo.com (H.H.); 3 Department of Life Sciences, Texas A&M Corpus Christi, 6300 Ocean Drive, Corpus Christi, TX 78412, USA; paul.zimba@tamucc.edu; 4 Emerging Toxins Program, National Ocean Service/NOAA, Hollings Marine Laboratory, 331 Fort Johnson Road, Charleston, SC 29412, USA; peter.moeller@noaa.gov

**Keywords:** *Trichodesmium thiebautii* blooms, polyketide, polyol, secondary metabolite

## Abstract

In an effort to isolate and characterize bioactive secondary metabolites from *Trichodesmium thiebautii* blooms, collected cyanobacteria biomass was subjected to bioassay-guided extraction and fractionation using the human colon cancer cell line HCT-116, resulting in the isolation and subsequent structure characterization of a linear polyketide trichophycin A (**1**). The planar structure of **1** was completed using 1D and 2D NMR spectroscopy and high-resolution electrospray ionization mass spectrometry (HRESIMS). Trichophycin A was moderately toxic against the murine neuroblastoma cell line Neuro-2A (EC_50_: 6.5 μM) and HCT-116 cells (EC_50_: 11.7 μM). Trichophycin A was significantly more cytotoxic than the previously isolated polyketides trichotoxin A and trichotoxin B. These cytotoxicity observations suggest that toxicity may be related to the polyol character of these polyketide compounds.

## 1. Introduction

Many biosynthetic systems in marine cyanobacteria allow for the incorporation of peptide and polyketide subunits resulting in remarkable structural diversity [1,2]. These resultant hybrid non-ribosomal peptide synthetase-polyketide synthase NRPS-PKS molecules have remained a frequently isolated secondary metabolite class from cyanobacteria [3] and they have shown diverse and therapeutically relevant biological activities [4,5,6,7]. Macrocyclic and linear polyketides have been isolated from cyanobacteria with less regularity than NRPS and NRPS-PKS molecules. Still, many of these polyketides have exhibited a diverse array of biological activities [8,9,10] and possess highly functionalized structural elements [11,12,13] demonstrating the importance of their continued isolation and biological evaluation.

*Trichodesmium thiebautii*, a marine filamentous cyanobacterium of the order *Oscillatoriales*, is globally significant both for its biogeochemical role in N_2_ fixation [14] and the biological community associated with the “floating islands” formed by dense blooms that can cover many kilometers of surface waters [15]. *Trichodesmium* blooms occur over most of the subtropical and tropical oceans with the greatest concentration in the eastern tropical Pacific Ocean and the Arabian Sea [16]. Repeated blooms occur in the eastern and western Gulf of Mexico from February through August [17]. These blooms have been hypothesized to contribute the nitrogen required for the initiation of *Karenia brevis* blooms in the Gulf of Mexico [18]. Studies carried out using homogenized cells, filtrates, aging cultures, and crude extracts of *Trichodesmium thiebautii* filaments have shown toxicity to copepods [19,20]. However, establishing uni-algal, actively growing cultures of *T. thiebautii* has been problematic and has limited the ability to characterize these toxic molecules [21]. Thus, members of the cyanobacterial genus *Trichodesmium* remain an under-sampled group with few metabolites described from the genus. The cyclic peptide trichamide was characterized from a cultured strain of *Trichodesmium erythraeum* [22]. The lipoamides, credneramides A and B, were isolated and characterized from a field-collected benthic cyanobacterium. This specimen showed a phylogenetic relationship to other *Trichodesmium* strains and may be a new species [23]. A chlorinated metabolite, trichotoxin, displaying some cytotoxicity was isolated from a field sample of *Trichodesmium thiebautii* [24]. Recently, our laboratory has revised the structure of trichotoxin, renaming it trichotoxin A, and have isolated and characterized an alkyne-containing analog trichotoxin B [25].

Herein, we report the isolation and structure characterization of trichophycin A (**1**), a linear triol polyketide. This molecule possesses, presumably, an aromatic biosynthetic starter unit, while the remainder of the molecule is recognizably derived from a PKS system, likely incorporating acetate extensions, the last of which we predict would undergo decarboxylation resulting in terminal olefin formation. Trichophycin A is structurally similar to the compounds trichotoxin A and B [25], which were also isolated from a *T. thiebautii* bloom. Trichophycin A (**1**) showed moderate cytotoxicity against Neuro-2A murine neuroblastoma cells and HCT-116 human colon cancer cells with EC_50_ values of 6.5 ± 1.4 μM and 11.7 ± 0.6 μM, respectively.

## 2. Results

### 2.1. Structure Elucidation of ***1***

**Figure 1:**
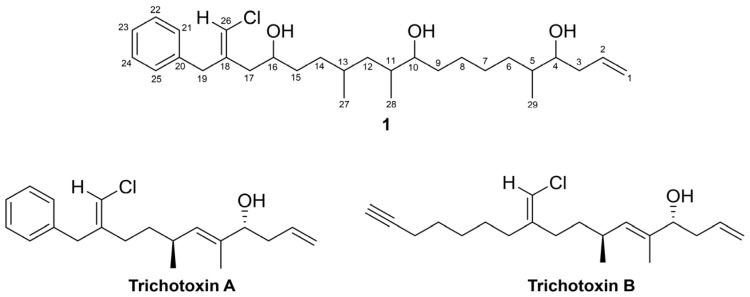
Structures of trichophycin A (**1**), trichotoxin A, and trichotoxin B.

Bioassay-guided fractionation of the *T. thiebautii* extract and chemical fractions against HCT-116 cells led to the isolation of **1** (Figure 1), an optically active pale yellow oil. HRESIMS analysis of **1** gave an [M + H]^+^ of *m/z* 479.3282, suggesting a molecular formula of C_29_H_47_ClO_3_ and a requirement of 6 degrees of unsaturation. Examination and comparison of the ^13^C-NMR, HSQC and HMBC spectra of **1** showed the presence of 3 methyl groups, 11 methylenes, 13 methines, and 2 quaternary carbon atoms (Figures S2–S4).

**Figure 2:**
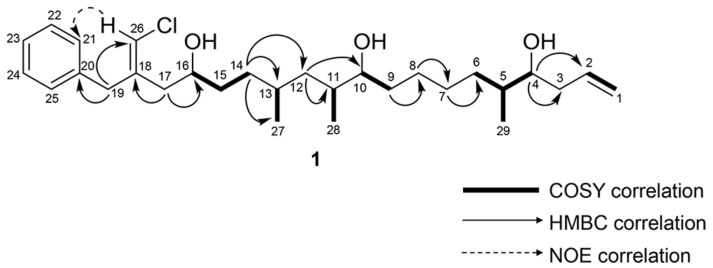
Key COSY, HMBC, and NOE correlations of **1**.

The ^13^C-NMR spectra of **1** showed the characteristic signals of a phenyl group for positions C-20 to C-25 (δ_C_ 138.0, 129.0, 128.6, 126.7, 128.6, 129.0). A deshielded methylene (H_2_-19, δ_H_ 3.46) showed HMBC correlations to the quaternary carbon of the aromatic ring (C-20, δ_C_ 138.0), and C-21 and C-25 on the aromatic ring (δ_C_ 129.0). Additionally, the H_2_-19 methylene showed HMBC correlations to a moderately polarized olefin comprised of a quaternary carbon (C-18, δ_C_ 139.5) and a carbon (C-26, δ_C_ 116.2) bearing a singlet methine proton at δ_H_ 5.99. These chemical shifts were consistent with the presence of a vinyl chloride functionality, and considering the aromatic moiety, accounted for 5 of the 6 degrees of unsaturation required. NOE correlations between the H-26 (δ_H_ 5.99) and H-21 (δ_H_ 7.17) and H_2_-19 (δ_H_ 3.46) supported a *Z* configuration of the vinyl chloride (Figure S7). The H_2_-19 methylene showed HMBC correlation to C-17 (δ_C_ 38.2). The HSQC showed C-17 was correlated to deshielded methylene protons (δ_H_ 2.38, 2.28). This second group of deshielded methylene protons (H_2_-17) showed HMBC correlations to the vinyl chloride-containing olefin and to an oxygen-bearing carbon (C-16, δ_C_ 70.7). Examination of the COSY spectrum (see Figure S5) showed that the oxymethine (H-16, δ_H_ 3.79) was coupled to a methylene group (H_2_-15 δ_H_ 1.52 and 1.44). These H_2_-15 protons showed COSY correlations to the H_2_-14 methylene (δ_H_ 1.35 and 1.22). The H_2_-14 methylene protons showed HMBC correlations to C-13 (δ_C_ 29.8), C-12 (δ_C_ 40.8), and the C-27 methyl group (δ_C_ 20.4). The H-13 methine (δ_H_ 1.52) showed a COSY correlation to H_3_-27 (δ_H_ 0.87). The H_2_-12 methylene protons (δ_H_ 1.39, 1.00) showed HMBC correlations to a second tertiary carbon (C-11, δ_C_ 35.2) bearing an adjacent methyl group (C-28, δ_C_ 13.9) and an HMBC correlation to a second oxygen-bearing carbon (C-10, δ_C_ 74.5). Bidirectional HMBC correlations established four methylene groups between C-10 and C-5. The H-5 methine showed COSY correlations to H-6b (δ_H_ 1.19), H_3_-29 (δ_H_ 0.90), and the H-4 oxymethine (δ_H_ 3.55). The oxymethine showed HMBC correlations to C-3 (δ_C_ 39.1) and C-2 (δ_C_ 135.6). A COSY correlation between H-2 (δ_H_ 5.83) and H_2_-1 (δ_H_ 5.15, 5.12) established a monosubstituted terminal olefin functionality, satisfied the final degree of unsaturation, and demonstrated that **1** was a linear polyketide. Selected 2D correlations utilized in determining the planar structure of **1** are shown in Figure 2. Examination of the TOCSY spectrum supported COSY correlations (Figure S6).

### 2.2. Cyotoxicity of Trichophycin A

**Figure 3:**
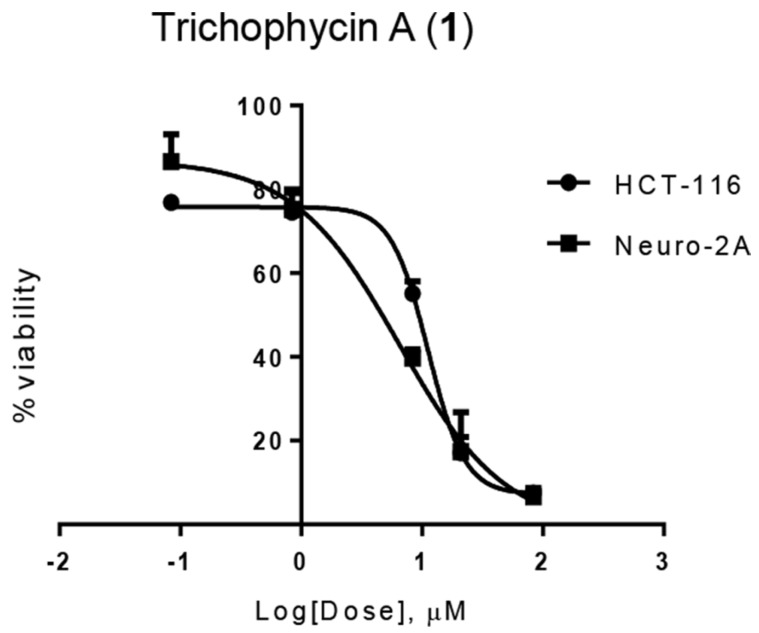
Dose-response curves of trichophycin A (**1**) tested against Neuro-2A murine neuroblastoma cells and HCT-116 human colon cancer cells.

Trichophycin A (**1**) showed moderate cytotoxicity against selected cells lines with EC_50_ values against Neuro-2A cells and HCT-116 cells of 6.5 ± 1.4 μM and 11.7 ± 0.6 μM, respectively (Figure 3). However, there was no significant selectivity between the cell lines tested (*t*-test, *p* > 0.05). The EC_50_ values for trichotoxins A and B were greater than 50 μM against Neuro-2A cells and greater than 100 μM against HCT-116 cells (See Figure S8).

## 3. Discussion

Trichophycin A (**1**) represents a new addition to the diverse group of vinyl chloride-containing linear polyketides and polyketide-peptides isolated from cyanobacteria environmental collections. This group includes the jamaicamides [26], the credneramides [23], the kimbeamides [11], coibacin C and D [12], and pitiamides A and B [27] among others. We would predict that the biosynthesis of the vinyl chloride in trichophycin A would follow that of the jamaicamides in which an acetate-derived vinyl group is generated through the action of a hydroxymethylglutaryl-CoA (HMG-CoA) synthase cassette and subsequently halogenated [26]. Trichophycin A shows several structural similarities to trichotoxin A and B [25]. All possess vinyl chloride functionalities and terminal alkenes. Trichophycin A (**1**) features a longer polyketide chain than trichotoxin A with two additional secondary alcohol groups and one additional branched methyl. This increased polyol character may be a reason for the increased cytotoxicity of **1** (EC_50_ = 6.5 μM) compared to that originally reported for trichotoxin A (LC_50_ = 106 μM) against Neuro-2A cells [24] and this study (EC_50_ > 50 μM against Neuro-2A cells). The number of free hydroxyl groups is critical to the antiproliferative effect of the macrocyclic polyketide amantelide A, as its peracetylated derivative was essentially inactive [9]. This effect was also observed with kalkipyrone B and its acetylated derivative [8]. These modified products have decreased hydroxyl character. However, the acetyl groups add steric bulk which may interfere with receptor binding. Testing a comprehensive series of analogs with decreasing and increasing hydroxyl character will ultimately provide a more informative structure-activity relationship (SAR) study Many of the previously mentioned vinyl chloride-containing compounds have shown neuromodulatory activity [11,23,26]. Trichophycin A (**1**) does show greater potency against Neuro-2A cells than HCT-116 cells. However, the difference in potency is not significant. Further evaluation of **1** against a diverse panel of eukaryotic cells will determine if trichophycin A is selective or if it is a broad-spectrum moderately toxic molecule. The amount of trichophycin A isolated (0.10% of dry weight of the cyanobacterial biomass) suggests this molecule may have an important ecological or physiological role for the organism. The complex methylene and methine envelope and overlapping signals from 1.20 to 1.70 ppm in the ^1^H-NMR spectrum (see Figure S1) was not amenable to analysis following derivatization of the secondary alcohol groups in **1** with chiral shift reagents. Thus, absolute configuration analysis will necessitate total synthesis, X-ray diffraction analysis of a crystallized derivative or bioinformatic analysis of the ketoreductase (KR) domains in the trichophycin biosynthetic gene cluster. Ketoreductases generally control the configuration of β-hydroxy and secondary methyl groups in polyketide biosynthesis [28,29,30]. Work will continue to explore potential biological activities of **1** and search for structurally related analogs from *Trichodesmium* blooms in order to continue to evaluate the relationship between polyol character and cytotoxicity.

## 4. Materials and Methods

### 4.1. General Experimental Procedures

Optical rotations were measured using a Jasco P-2000 polarimeter (Jasco Inc., Easton, MD, USA). UV spectra were measured using a Beckman Coulter DU-800 spectrophotometer (Beckman Coulter Inc., Brea, CA, USA). NMR spectra were collected using a Bruker 800 MHz NMR instrument (Bruker, Rheinstetten, Germany) equipped with a cryoprobe. HRESIMS analysis was performed using a AB SCIEX TripleTOF 4600 mass spectrometer (SCIEX, Framingham, MA, USA) with Analyst TF software (version 1.7, SCIEX, Framingham, MA, USA). Semi-preparative HPLC was carried out using an Agilent 1100 series system equipped with a micro vacuum degasser, an autosampler, and a diode-array detector.

### 4.2. Biological Material

A localized bloom of *Trichodesmium* was collected from Padre Island, Corpus Christi, TX during 9–11 May 2014. Surface bloom material was collected in 5-gallon buckets from ca. 0.5-m water depth. Approximately 300 g wet weight cell mass was concentrated from this material and frozen for further chemical analysis. In the laboratory, a subsample of the cell mass was examined microscopically and identified according to Komarek [31]. A preserved voucher sample of the biological material is kept in our laboratory with the identification number TTPI2014.

### 4.3. Extraction and Isolation of ***1***

*Trichodesmium thiebautii* filaments (14.4 g, dry weight), originally collected from a bloom near Padre Island, Corpus Christi, Texas in May of 2014, were extracted with five separate portions of 2:1 CH_2_Cl_2_-CH_3_OH resulting in 3.95 g of crude extract. The crude residue was reconstituted in hexanes and fractionated over silica gel using vacuum liquid chromatography (VLC) using a stepped gradient of hexanes, EtOAc, and CH_3_OH. The fractions eluting with 60% EtOAc in hexanes and 80% EtOAc in hexanes (Fractions E and F) were combined based on similarities in ^1^H-NMR signals and similar potency in cytotoxicity assays. The combined material was fractionated over a 2 g Strata C18 SPE column eluting with 50% CH_3_CN in H_2_O, 100% CH_3_CN, 100% CH_3_OH, and 100% EtOAc. The fraction eluting with CH_3_CN showed the most potent cytotoxicity and was subjected to reversed-phase HPLC using a YMC 5 μm ODS column (250 × 4.6 mm) with an elution solvent of 80% CH_3_CN in H_2_O with 0.1% formic acid added and trichophycin (**1**) (15 mg, rt: 9.25 min) was isolated.

*Trichophycin A* (**1**): pale yellow oil; $\alpha_{D}^{25}$ 4.3 (MeOH, *c* 0.20); UV (MeOH) λ_max_ (log ε) 204 (4.0) nm; ^1^H-NMR (800 MHz, CDCl_3_); and ^13^C-NMR (200 MHz, CDCl_3_), see Table 1; HRESIMS *m/z* 479.3282 [M + H]^+^ (calculated for C_29_H_48_ClO_3_, 479.3292).

### 4.4. Cytotoxicity Assays

HCT-116 cells were added to 96-well plates in 100 μL of McCoy’s 5A media at a density of 2000 cells/well. Neuro-2A cells were added to assay plates in 100 μL of Eagle’s Minimum Essential Media (EMEM) supplemented with 10% fetal bovine serum at a density of 5000 cells/well. Cells were incubated overnight (37 °C, 5% CO_2_) and examined microscopically to confirm confluence and adherence. Impure fractions were dissolved in CH_3_OH (1% *v*/*v*) and tested at concentrations of 40 and 4 μg/mL. Purified trichophycin A was dissolved in DMSO (1% *v*/*v*) and added to the cells in the range of 100, 10, 1, 0.1, and 0.01 μM in order to construct a dose response curve. Three technical replicates were prepared for each concentration and each assay was performed in triplicate. Paclitaxel was used as a positive control (EC_50_ = 3.1 nM against HCT-116 cells; EC_50_ = 15.8 nM against Neuro-2A cells) and DMSO (1% *v*/*v*) was used as a negative control. Trichotoxin A and B were tested in the same manner. However, the concentrations tested were between 1 and 160 μM. Trichotoxin A and B assays were run in duplicate. Plates were incubated for 72 h after which 15 μL of MTT (3-(4,5-dimethylthiazol-2-yl)-2,5-diphenyltetrazolium) dye were added each assay well. The dye was allowed to incubate with the cells for 4 h after which media was aspirated and the remaining formazan crystals were solubilized in 100 μL of DMSO. Absorbance at 570 nm was measured using a Molecular Devices SpectraMax plate reader and EC_50_ curves were generated and statistical procedures were performed using Graphpad Prism software.

## Figures and Tables

**Table 1:** NMR data for trichophycin A (**1**) (800 MHz, CDCl_3_ for ^1^H-NMR).

Pos	δ_C_, Type	δ_H_ (*J* in Hz)	HMBC	COSY	NOESY
1a	117.9, CH_2_	5.15, m	2, 3	2	
1b		5.12, m	2, 3	2	
2	135.6, CH	5.83, m	3, 4	1a, 1b, 3a, 3b	
3a	39.1, CH_2_	2.26, m	1, 2, 4, 5	2, 3b, 4	29
3b		2.16, m	1, 2, 4, 5	2, 3a, 4	6b, 29
4	73.9, CH	3.55, dt (8.9, 4.0)	2, 3, 5, 6, 29	3a, 3b, 5	6b, 7b, 29
5	37.8, CH	1.53, ovlp ^a^	4, 29	4, 6b, 29	3a, 3b
6a	33.0, CH_2_	1.48, m	4, 5, 7, 8, 29	6b, 7a, 7b	3b
6b		1.19, m	4, 5, 7, 8, 29	5, 6a	3b, 4
7a	27.4, CH_2_	1.38, ovlp	5, 6, 8		
7b		1.32, ovlp	6, 8		4
8a	26.7, CH_2_	1.44, ovlp	7, 9		
8b		1.32, ovlp	6, 7, 10		
9	34.6, CH_2_	1.44, ovlp	10		10
10	74.5, CH	3.50, m	8, 9, 11, 12, 28	9, 11	12b, 13, 28
11	35.2, CH	1.59, m	9, 10, 12, 13, 28	10, 12a, 12b, 28	
12a	40.8, CH_2_	1.39, ovlp	10, 11, 13, 14 27, 28	12b	
12b		1.00, m	10, 11, 13, 14, 27, 28	11, 12a	10
13	29.8, CH	1.52, ovlp	12, 14, 27	12b, 14b, 27	10, 16
14a	32.5, CH_2_	1.35, ovlp	12, 13, 15, 16, 27	14b, 15b	16, 17b
14b		1.22, m	12, 13, 15, 16, 27	14a, 15b	16, 17b
15a	34.8, CH_2_	1.52, ovlp	13, 14, 16, 17	14a, 14b, 16	17a, 17b
15b		1.44, ovlp	13, 14, 16, 17	14b, 16	17a, 17b
16	70.7, CH	3.79, m	14, 15, 17, 18	15a, 15b, 17a, 17b	4a, 13, 14b, 27
17a	38.2, CH2	2.38, dd (13.7, 8.8)	15, 16, 18, 19, 26	16, 17b	15a, 15b, 19
17b		2.28, dd (13.7, 4.2)	15, 16, 18, 19, 26	16, 17a	15a, 15b, 19
18	139.5, qC				
19	41.9, CH_2_	3.46, d (5.5)	17, 18, 20, 21, 25, 26		17a, 17b, 26
20	138.0, qC				
21	129.0, CH	7.17, d (7.6)	19, 22, 23	22	19, 26
22	128.6, CH	7.30, t (7.6)	20	21	
23	126.7, CH	7.23, t (7.6)	22, 24	22, 24	
24	128.6, CH	7.30, t (7.6)	20	25	
25	129.0, CH	7.17, d (7.6)	19, 23, 24	24	19
26	116.2, CH	5.99, s	16, 17, 18, 19		19, 21
27	20.4, CH_3_	0.87, d (6.6)	12, 13, 14	13	10, 11, 14b, 16
28	13.9, CH_3_	0.84, d (6.8)	10, 11, 12	11	9, 10
29	13.9, CH_3_	0.90, d (6.8)	4, 5, 6	5	3a, 3b, 4, 6b

^a^ overlapping signals.

## Supplementary Materials

The following are available online at www.mdpi.com/1660-3397/15/1/10/s1, Figures S1–S7: 1H-NMR, 13C-NMR, HSQC, HMBC, COSY, TOCSY, NOESY data for compound **1**. Figure S8: Cytotoxicity data of trichotoxin A and B against HCT-116 cells.
