# Supplementary material for: Trichophycin A, a Cytotoxic Linear Polyketide Isolated from a Trichodesmium thiebautii Bloom
Source: Mar Drugs. 2017 Jan 6;15(1):10. doi: 10.3390/md15010010 (PMC5295230; doi:10.3390/md15010010)
Supplement: Supplementary file 1 [file marinedrugs-15-00010-s001.docx]

Supplementary Materials: Trichophycin A,
a Cytotoxic Linear Polyketide Isolated from a *Trichodesmium thiebautii* Bloom

Matthew J. Bertin, Paul G. Wahome, Paul V. Zimba, Haiyin He and Peter D. R. Moeller

**Figure S1.** ^1^H-NMR spectrum of trichophycin A (**1**).

**Figure S2.** ^13^C-NMR spectrum of **1**.

**Figure S3.** HSQC spectrum of **1**.

**Figure S4.** HMBC spectrum of **1**.

**Figure S5.** COSY spectrum of **1**.

**Figure S6.** TOCSY spectrum of **1**.

**Figure S7.** NOESY spectrum of **1**.

**Figure S8.** Cytotoxicity of trichotoxin A and trichotoxin B against HCT-116 cells.


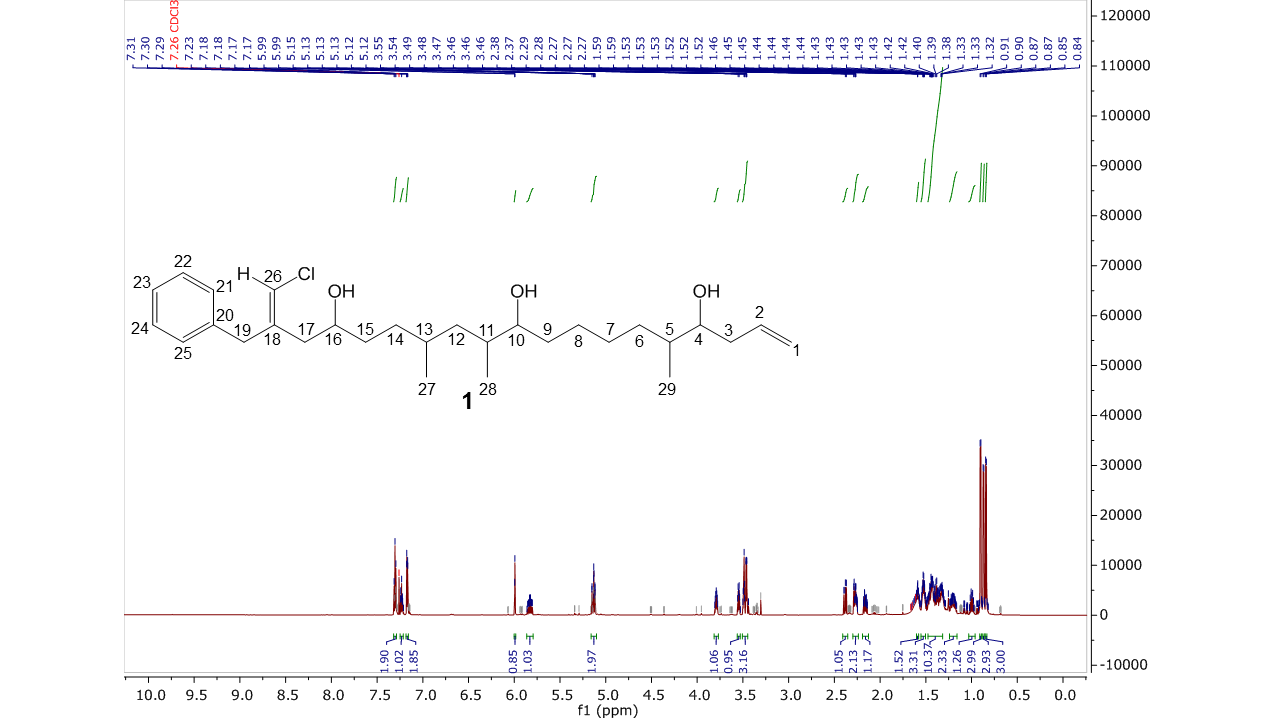


**Figure S1.** ^1^H-NMR spectrum of trichophycin (**1**).


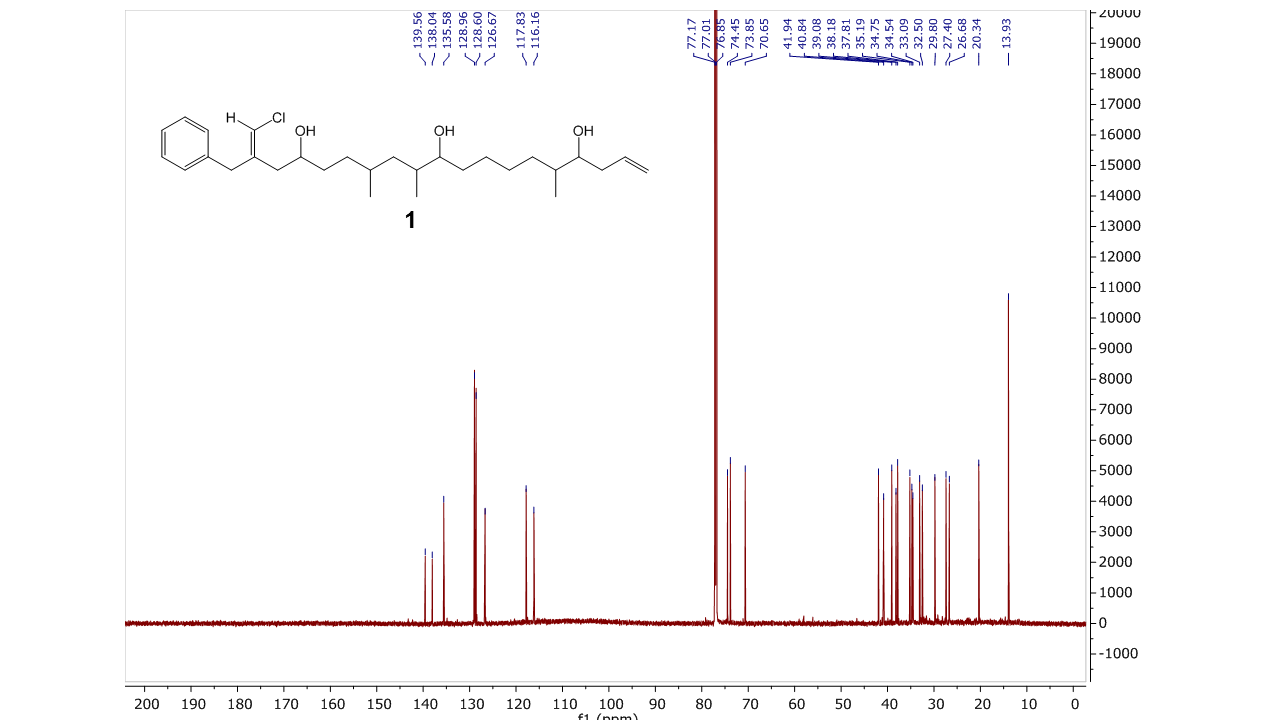


**Figure S2.** ^13^C-NMR spectrum of **1**.


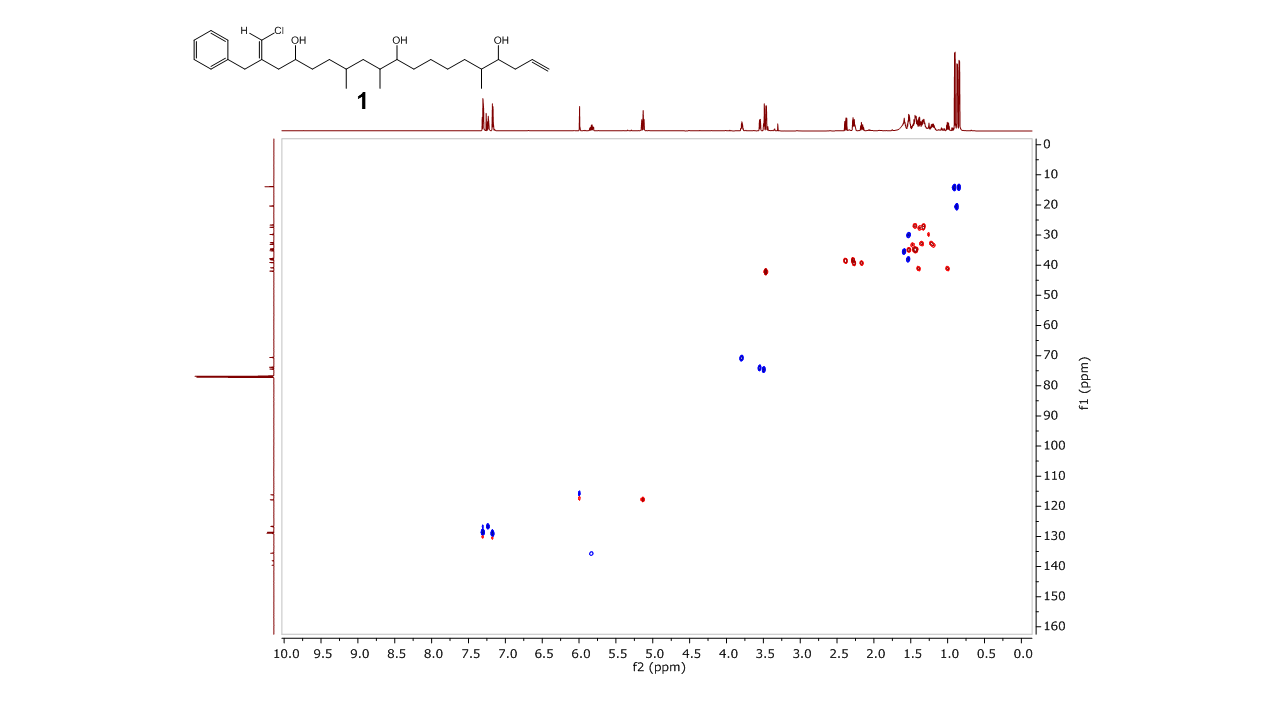


**Figure S3.** HSQC spectrum of **1**.


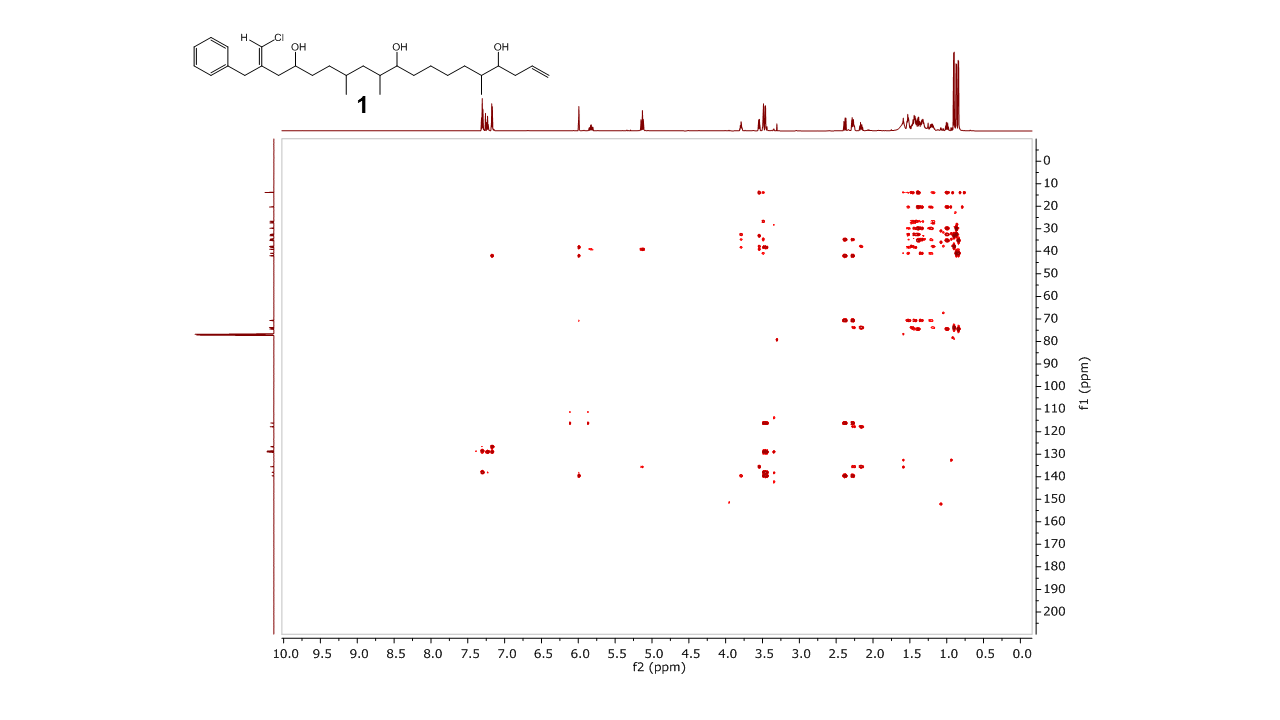


**Figure S4.** HMBC spectrum of **1**.


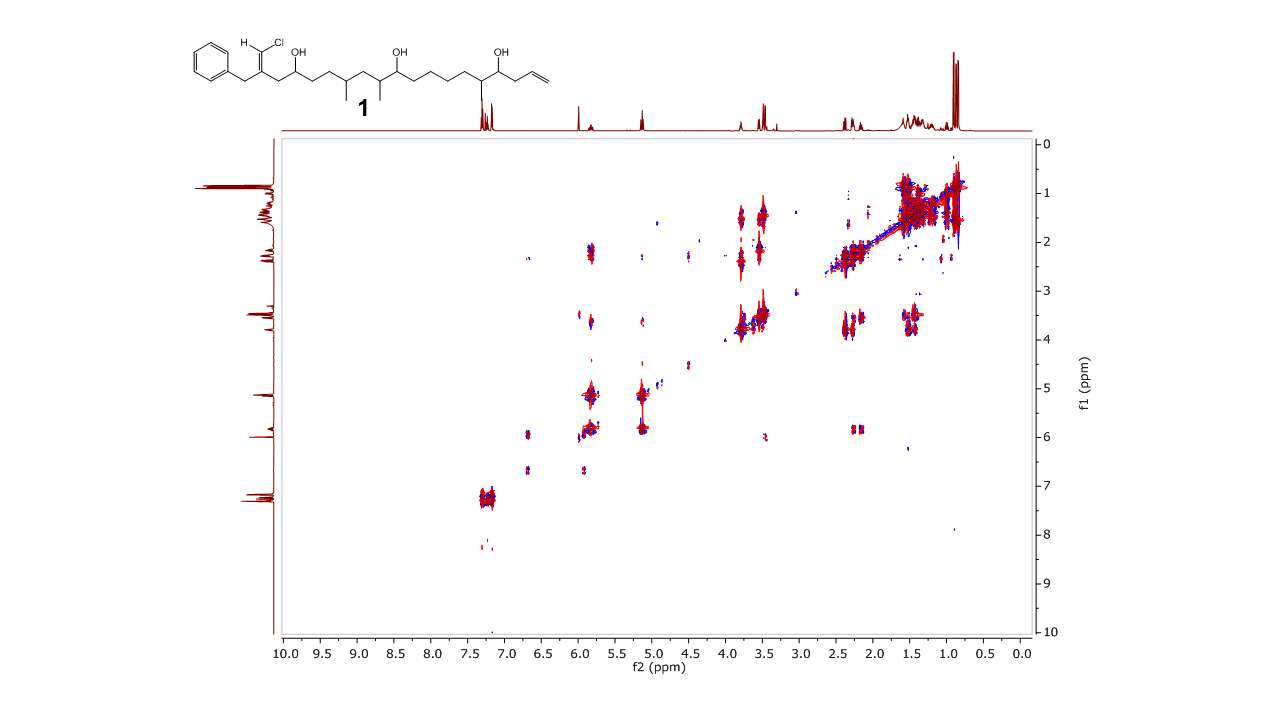


**Figure S5.** COSY spectrum of **1**.


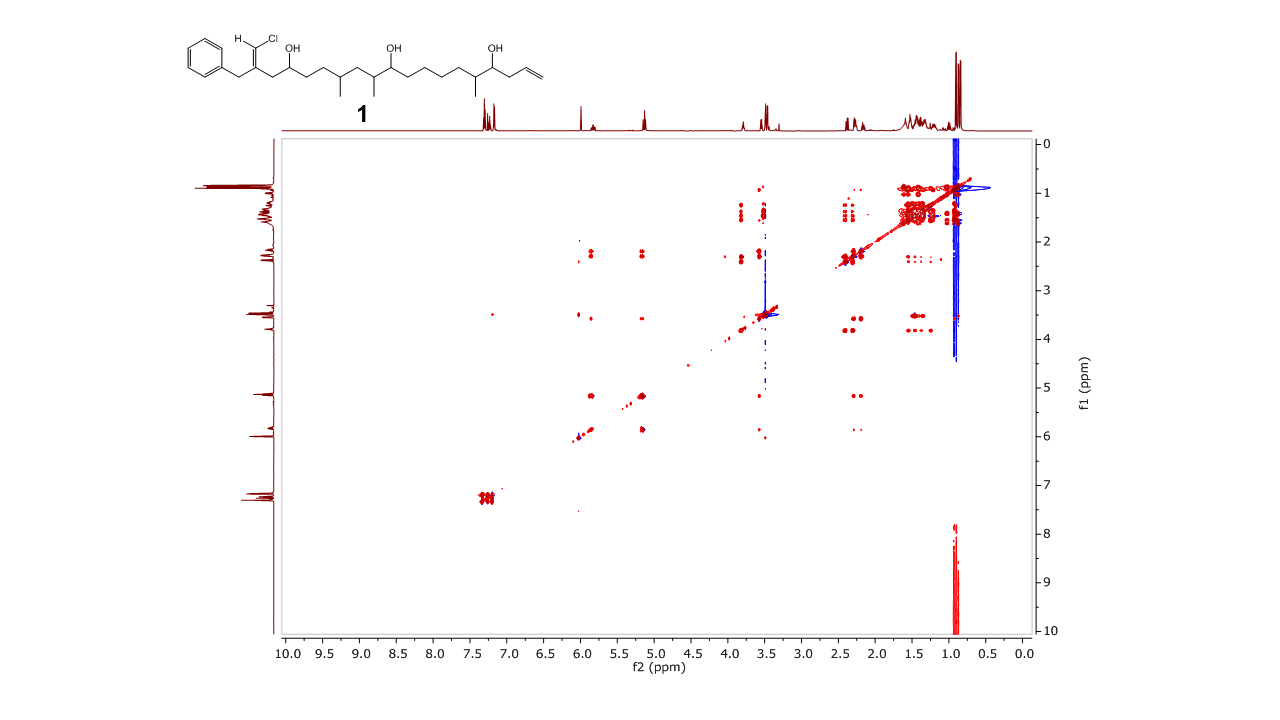


**Figure S6.** TOCSY spectrum of **1**.


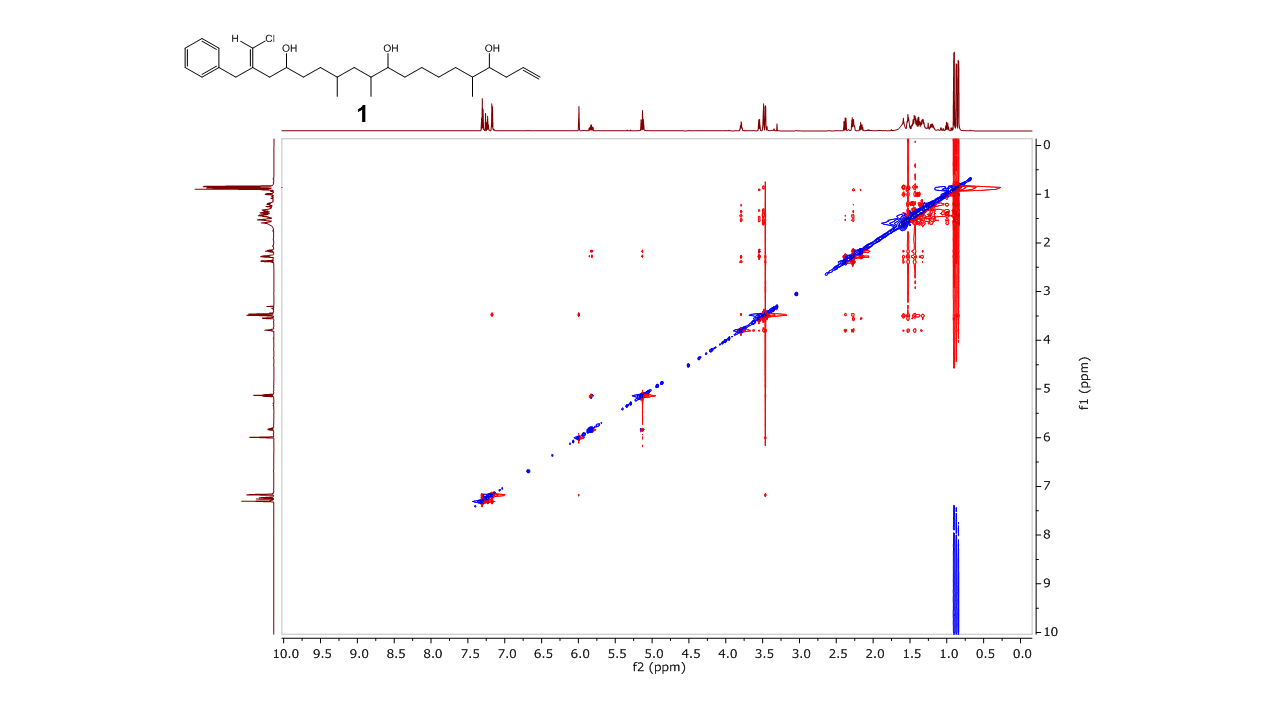


**Figure S7.** NOESY spectrum of **1**.


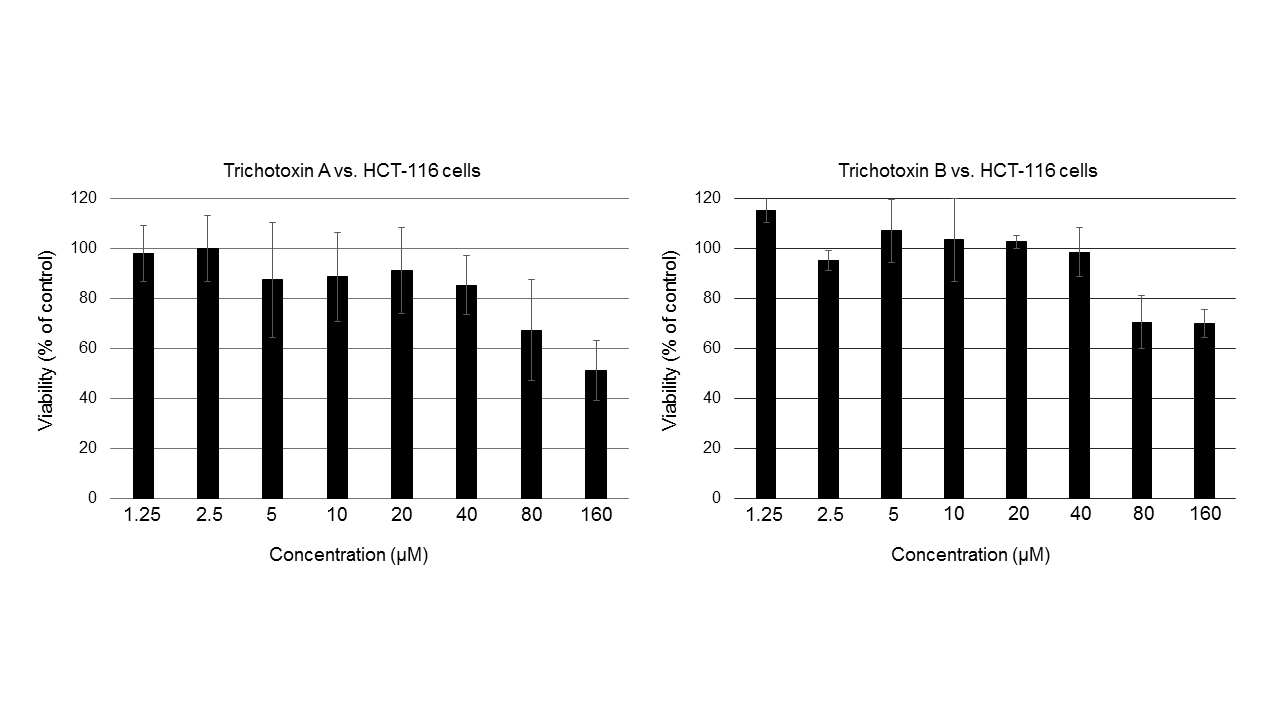


**Figure S8.** Cytotoxicity of trichotoxin A and trichotoxin B against HCT-116 cells. Bars represent the average % viability (compared to negative control) of four technical replicate wells ± SD. Experiments were conducted in duplicate.
